# Exposure to Cigarette Smoke Enhances Pneumococcal Transmission Among Littermates in an Infant Mouse Model

**DOI:** 10.3389/fcimb.2021.651495

**Published:** 2021-03-31

**Authors:** Daichi Murakami, Masamitsu Kono, Denisa Nanushaj, Fumie Kaneko, Tonia Zangari, Yasuteru Muragaki, Jeffrey N. Weiser, Muneki Hotomi

**Affiliations:** ^1^ Department of Otorhinolaryngology-Head and Neck Surgery, Wakayama Medical University, Wakayama, Japan; ^2^ Department of Otolaryngology, Tokyo Women’s Medical University Medical Center East, Tokyo, Japan; ^3^ Department of Microbiology, New York University School of Medicine, New York, NY, United States; ^4^ Department of Pathology, Wakayama Medical University, Wakayama, Japan

**Keywords:** *Streptococcus pneumoniae*, transmission, cigarette smoke, children, mouse model

## Abstract

*Streptococcus pneumoniae*, one of the most common commensal pathogens among children, is spread by close contact in daycare centers or within a family. Host innate immune responses and bacterial virulence factors promote pneumococcal transmission. However, investigations into the effects of environmental factors on transmission have been limited. Passive smoking, a great concern for children’s health, has been reported to exacerbate pneumococcal diseases. Here, we describe the effect of cigarette smoke exposure on an infant mouse model of pneumococcal transmission. Our findings reveal that the effect of cigarette smoke exposure significantly promotes pneumococcal transmission by enhancing bacterial shedding from the colonized host and by increasing susceptibility to pneumococcal colonization in the new host, both of which are critical steps of transmission. Local inflammation, followed by mucosal changes (such as mucus hypersecretion and disruption of the mucosal barrier), are important underlying mechanisms for promotion of transmission by smoke exposure. These effects were attributable to the constituents of cigarette smoke rather than smoke itself. These findings provide the first experimental evidence of the impact of environmental factors on pneumococcal transmission and the mechanism of pathogenesis.

## Introduction

*Streptococcus pneumoniae* (*Sp*.; the pneumococcus) is one of the leading pathogens responsible for upper respiratory infections or invasive infections during childhood. When pneumococcus moves to body sites that are typically sterile it can cause various diseases (otitis media, rhinosinusitis, pneumonia, meningitis, and sepsis). *S. pneumoniae* can transmit from host to host by close contact with respiratory secretions, which often occurs among families or in daycare centers. Despite near-global use of pneumococcal vaccines, pneumococcal diseases remain among those of greatest medical concerns for children and the elderly. There is a need to investigate mechanisms involved in transmission and to develop preventive strategies against pneumococcal colonization, the first step in disease.

Important steps for pneumococcal transmission are exit from a colonized host (shedding) and acquisition by a new host (colonization) (Weiser et al., 2018). Animal studies have revealed that the induction of inflammation in the nasal cavity of a *Sp.*-colonized host increases bacterial shedding, resulting in an increase in pneumococcal transmission. Co-infection with influenza A virus (IAV) dramatically increases transmission of the pneumococcus by increasing secretions (which carry *Sp.* to a new host) due to increased nasopharyngeal inflammation (Short et al., 2012; Richard et al., 2014). Host innate immunity factors such as TLR2 and TLR3 were shown to be involved in the mechanism of increased pneumococcal transmission in an IAV co-infection model (Richard et al., 2014; Kono et al., 2016). In the infant mouse model, pneumococcal mono-infection caused a subtle but acute inflammatory response which resulted in pneumococcal shedding and transmission among littermates (Zafar et al., 2016). Pneumolysin, a pivotal pneumococcal virulence factor, contributes not only to pneumococcal colonization density in infant mice but also promotes shedding and transmission due to local inflammation caused by tissue damage (Hotomi et al., 2016; Zafar et al., 2017).

Despite the progress of research on host immunity and pathogens, there is little understanding of the environmental factors that promote pneumococcal transmission. Smoking has long been one of the major health problems in the world: according to the World Health Organization, smoking causes more than 7 million deaths every year (World Health Organization, 2017). Passive smoking or second-hand smoking, defined as involuntary exposure to smoke formed from the burning of cigarettes and smoke exhaled by the smoker (Oberg et al., 2010), significantly elevates the risk for bacterial diseases among children, including invasive pneumococcal disease (Nuorti et al., 2000), middle ear disease, and lower respiratory tract infections (Cao et al., 2015). Some *in vivo* studies have shown that cigarette smoke exposure promotes nasopharyngeal colonization or invasive infection of pneumococci in an adult animal model (Voss et al., 2015; Shen et al., 2016), but the underlying mechanism is not fully understood. Additionally, there are no studies modeling the impact of passive smoking on pneumococcal colonization and transmission during early childhood. It is known that cigarette smoke causes local inflammation and injury of respiratory tissue accompanied by an influx of neutrophils and macrophages (Jaspers, 2014; Strzelak et al., 2018). Inflammation could promote pneumococcal growth and injury could inhibit clearance of the bacteria from the mucosal surface. Cigarette smoke extract (CSE) has been recently used as an alternative to actual cigarette smoking in the laboratory setting (Elliott et al., 2006; Mizutani et al., 2009; Wu et al., 2014; Ueha et al., 2016; Jia et al., 2019). CSE is made by bubbling cigarette smoke through saline, and is reported to have approximately equivalent effect to cigarette smoke on the subjects which received the CSE (Mizutani et al., 2009; Ueha et al., 2016). In the current study, we provided CSE by intranasal administration to model a passive smoking state among the infant mice, thereby also avoiding the confounding effects of maternal exposure to cigarette smoke. We hypothesized that passive smoking would increase pneumococcal colonization and transmission by causing nasopharyngeal inflammation and disruption of the mucosal barrier.

## Materials and Methods

### Ethics Statement

This study was conducted according to the guidelines outlined by National Science Foundation Animal Welfare Requirements and the Public Health Service Policy on the Humane Care and Use of Laboratory Animals. This project was approved by the Institutional Animal Care and Use Committee at Wakayama Medical University (approved number: 882).

### Bacterial Strain and Growth Conditions

P2431, a serotype 6A *S. pneumoniae* (*Sp.*) strain resistant to streptomycin was used in all experiments. It is already known that transmission ratio varies depending on serotype (Zafar et al., 2016). To investigate the promoting effect on transmission by CSE, we selected serotype 6A which is unlikely to transmit comparing to other serotypes. The bacteria were grown in tryptic soy (TS) broth (Becton Dickinson (BD), Franklin Lakes, NJ) to mid-exponential phase at 37°C. When the bacterial culture reached the desired optical density (OD) at 600 nm, bacteria were washed and diluted in sterile phosphate-buffered saline (PBS) for inoculation. Quantitative culture was performed by plating 10-fold serial dilutions in triplicate on TS agar plates containing streptomycin (200 µg/ml) and catalase (6,300 U/plate) (Worthington Biochemical Corporation, Lakewood, NJ). Plates were incubated overnight at 37°C + 5% CO_2_. Bacterial stocks were stored in 20% glycerol at -80°C.

### Mice

Pregnant female wildtype C57BL/6 mice were obtained from Charles River Laboratories Japan, INC (Yokohama, Japan). Mice were maintained in a conventional animal facility and pups remained with their dam for the course of the experiment. Pups inoculated intranasally with chemical or infectious agents were monitored throughout the duration of the experiments and appeared healthy and gained weight similar to untreated animals.

### Cigarette Smoke Extract (CSE)

CSE was obtained from CMIC Pharma Science Co., Ltd (Yamanashi, Japan). Briefly, CSE is prepared by bubbling a stream of smoke from 50 of Hi-Light cigarettes (Japan Tobacco Inc., Tokyo) through 50ml of saline (Mizutani et al., 2009). For standardization, CSE was diluted 100-fold, and an OD at 267 nm was used as the indicator of concentration; the standardized concentration was adjusted to ~1.308. Undiluted CSE was used for all experiments similarly as previous reports (Mizutani et al., 2009; Ueha et al., 2016; Ueha et al., 2017). CSE was stored at -80°C until use.

### Infant Mouse Model of Cigarette Smoke Exposure and Infection

From day 4-7 of life, pups were given CSE (3µl/mouse), intranasally, twice a day without anesthesia; pups in the control group received 3µl PBS. On day 8 of life, pups were inoculated intranasally with 8,000 CFU of *S. pneumoniae* suspended in 3µl of PBS, without anesthesia.

### Evaluation of Nasal Colonization and Shedding

The pneumococcal nasal colonization levels were measured by enumerating colonies in a nasal wash or a nasal tissue. Pups were euthanized with isoflurane and the upper respiratory tract was lavaged with 200µl of sterile PBS from a 25G needle inserted in the trachea; lavages were collected from the nares. After washing nasal cavity, the nasal tissue was homogenized in 1ml of sterile PBS. The nasal lavages or homogenized tissues were serially diluted and plated on TS agar plates containing streptomycin (200 µg/ml) and catalase (6,300 U/plate) and incubated overnight at 37°C + 5% CO_2_. The pneumococcal colonies were counted to determine the colonization in each mouse. The limit of detection was 666 CFU/ml.

To assess pneumococcal shedding, from day 1 to 4 p.i., daily nasal secretions were collected by gently tapping (10 times) the nares onto TS agar plates containing streptomycin (200 µg/ml) and catalase (6,300 U/plate) as reported previously (Richard et al., 2014; Kono et al., 2016; Zafar et al., 2016; Zafar et al., 2017). The sample was then evenly spread across the plate using a sterile swab and incubated overnight at 37°C + 5% CO_2_ for quantitative culture.

### Mouse Model of Pneumococcal Transmission

The transmission model was modified for the current study (Richard et al., 2014; Kono et al., 2016). First, all pups were treated intranasally with 3µl of CSE (“index-and-contact CSE” group) or 3µl of PBS (“index-and-contact PBS” group) from days 4-7 of life as described above. Then half of the pups in a litter were intranasally inoculated with *Sp.* (8,000 CFU) at day 8 of life (‘index mice’). The pups were then returned to the dam and their uninfected littermates (‘contact mice’). At day 4 p.i. (day 12 of life), all pups were euthanized by isoflurane and the nasal lavages were collected. A transmission event was defined as the presence of the pneumococcus in the nasal wash of contact mice.

In other transmission experiments, only index or only contact pups received CSE (“index-CSE” and “contact-CSE” groups). In index-CSE groups, index mice received intranasal CSE and the contact mice received PBS; in contact-CSE groups, contact mice received intranasal CSE and the index mice received PBS. The litters were infected with *Sp.* and transmission was assessed as described above.

### Acquisition Model

After the pre-treatment with CSE or PBS on days 4-7 of life, pups were inoculated intranasally with a low dose of *S. pneumoniae* (~1,000 CFU) on day 8 of life. All pups were euthanized 7 or 24 hours after infection; nasal lavage was collected and pneumococcal colonization was determined as described as above.

### Histological Analyses

Pups were pre-treated with CSE or PBS and infected with *Sp.* as described above. On day 2 p.i. (day 10 of life), all pups were euthanized. The heads were removed and fixed in 4% paraformaldehyde for 2 days, then decalcified by ethylenediaminetetraacetic acid (EDTA) for 2 weeks. After decalcification, the tissue was dehydrated and embedded into paraffin. Histological analysis was performed with coronal sections. Tissues were sectioned with a thickness of 3µm at the nasal cavity and stained with hematoxylin and eosin (HE). Tissue samples for immunohistochemistry (IHC) double staining of type 6A pneumococcal capsule polysaccharide and Alcian blue for mucus were sectioned with a thickness of 4µm. After the activation of antigen for 20 min at 95°C, samples were incubated with rabbit antisera against type 6A pneumococcal polysaccharide (Statens Serum Institut, Denmark, 1:5,000 diluted in Dako REAL Antibody Diluent (Dako, Santa Clara, CA)) for 30 min at room temperature, and then incubated with un-diluted anti-rabbit secondary antibody (Histofine Simple Stain MAX PO (R), Nichirei Bioscience Inc., Tokyo, Japan) for 30 min at room temperature. In order to develop the color, samples were incubated with diaminobenzidine tetrahydrochloride (DAB) for 5 min at room temperature. The sections were then stained with Alcian blue solution (FUJIFILM Wako Pure Chemical Corporation, Osaka, Japan) for 30 min at room temperature and nuclei were stained by kernechtrot (MUTO PURE CHEMICALS CO., LTD., Tokyo, Japan) for 5 min at room temperature. Images were captured using KEYENCE BZ-X810 All-in-One Fluorescene Microscope (KEYENCE Japan, Osaka, Japan).

### Flow Cytometry

Neutrophils in nasal lavages were stained as previously described (Kono et al., 2016; Zafar et al., 2017). Pups exposed to CSE or PBS were pre-treated and infected with 8,000 CFU *Sp.* as described above. Pups were euthanized on day 2 or 4 p.i. and nasal lavages (200 µl) were collected. Pellets of nasal lavages were resuspended with PBS + 1% bovine serum albumin. Cells were stained with a 1:150 dilution of the following antibodies: anti-CD11b-V450 (BD), anti-Ly6G-PerCP-Cy (BD), and anti-CD45-APC-Cy7 (BD) for 30 min on ice in the dark after FcR blocking with a 1:200 dilution of anti-CD16/32 (BioLegend) for 15 min on ice. Cells were then fixed with 4% paraformaldehyde until analysis on FACS Verse (BD). Neutrophils were detected as CD11b+, Ly-6G+, CD45+ events.

### Immunoblot

After the pre-treatment of CSE or PBS, pups were intranasally inoculated with ~1,000 CFU and euthanized 24 hours post-infection. Nasal lavage samples (200µl) were 3-fold serially diluted and 300µl of each dilution was applied to a nitrocellulose membrane (Amersham Protran NC 0.2, Cytiva, Tokyo, Japan) with a slot-blot vacuum apparatus. The membranes were blocked with PBS containing 1% BSA, then probed with 1:500 dilution of the biotin-labeled Maackia Amurensis Lectin II (MAL II) (Vector Laboratories, Burlingame, CA) and then probed with 1:100,000 dilution of streptavidin conjugated to horseradish peroxidase (Abcam plc, Cambridge, UK) for 1 hour each, at room temperature. Immuno-reactive bands were visualized by chemiluminescence (ECL Prime Western Blotting Detection Reagent, Cytiva) and detected by imaging apparatus (LuminoGraph II, ATTO, Tokyo, Japan). The relative intensities of the bands on the immunoblot were quantified by measuring the integrated pixel density (IPD) using Adobe Photoshop (21.1.0 Release). For the purpose of analysis, the color of the blot was inverted so the background was black and the bands were white. An ellipse was drawn to encompass a band and the measurement was recorded. The same ellipse was used to measure all bands on the blot. A blank ellipse on the blot was measured to provide a background value that was subtracted from all other band IPD values.

### Statistical Analyses

Mann-Whitney U test or Fisher’s exact test was used for comparisons between two groups using GraphPad Prism 7 (GraphPad Software Inc., SanDiego, CA). Differences with *P* values of < 0.05 were considered statistically significant.

## Results

### Cigarette Smoke Exposure Increases Pneumococcal Colonization in the Upper Respiratory Tract of Infant Mice

**Figure 1:**
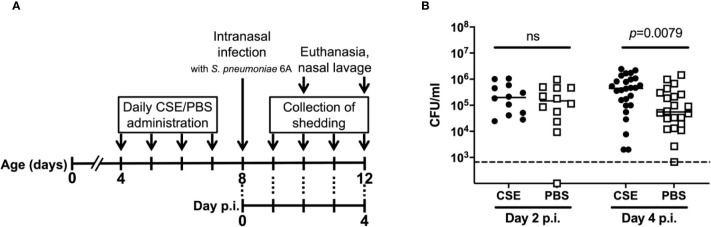
General experimental schematic for pneumococcal shedding and nasal colonization with CSE treatment. **(A)** Schematic of the experimental schedule. From day 4-7 of life, pups were inoculated intranasally with either CSE or PBS twice a day. At day 8 of life, pups were inoculated intranasally with *S. pneumoniae* 6A. Pups were euthanized on day 2 or 4 p.i (day 10 or 12 of life) and the pneumococcal burden obtained from nasal lavages was evaluated. Unless otherwise noted, the experiments were performed in this schedule. **(B)** Nasopharyngeal pneumococcal colonization assessed by nasal lavages. Bar represents the median values and each symbol represents the CFU/ml from a single mouse. CSE group (black circle) (*n* = 12 in Day 2 p.i. and 26 in Day 4 p.i.) and PBS group (open square) (*n* = 12 in Day 2 p.i. and 22 in Day 4 p.i.). Dotted line indicates the limit of detection (666 CFU/ml). Mann-Whitney U test was used for the statistical analyses.

To determine how cigarette smoke exposure affects pneumococcal colonization of the nasopharynx, neonatal mice were daily inoculated intranasally with 3µl of CSE or 3µl of phosphate buffered saline (PBS) from day 4-7 of life and on day 8 of life challenged with 8,000 CFU of *Sp*. Type 6A; pneumococcal colonization in the nasopharynx was assessed on day 10 or 12 of life. The experimental schematic is shown in **Figure 1A**. The density of nasal colonization was significantly higher in the CSE than the PBS control group 4 days post-infection (*p*=0.0079), although there was no difference between the groups on day 2 p.i. (**Figure 1B**). We also examined pneumococcal colonization in nasal tissue, but there were not any significant differences between the CSE and PBS groups (data not shown). These results suggest that passive smoking increases colonization density or the number of bacteria attaching to the epithelial surface rather than contributing to an increased number of bacteria penetrating the nasal subepithelial tissue.

### Cigarette Smoke Exposure Increases Pneumococcal Shedding and Neutrophil Influx Into the Nasal Cavity in Colonized Pups

**Figure 2:**
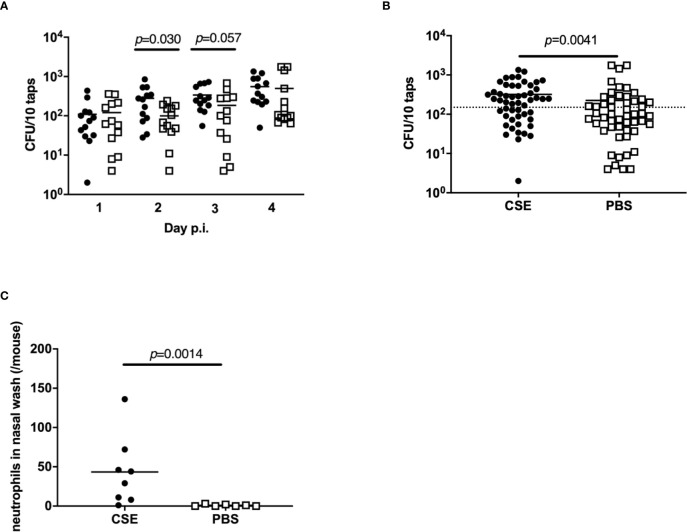
Bacterial shedding in nasal secretions and local neutrophil influx into the nasal cavity. Quantification of daily **(A)** and total (all 4 days) **(B)** bacterial shedding. Shedding values of each day post-infection; median indicated by the bar and each symbol represents the CFU/10 taps from a single mouse on a single day (*n* = 13 in each group). Dotted line indicates 150 CFU/10 taps. **(C)** Neutrophils in the nasal lavages quantified by flow cytometry. CSE group (black circle) (*n* = 8) and PBS group (open square) (*n* = 7). Mann-Whitney U test was used for the statistical analyses.

As the number of bacteria shed by colonized pups is one of critical factors impacting transmission, the effect of cigarette smoke exposure on pneumococcal shedding was evaluated. After pre-treatment of with CSE or PBS on days 4-7 of life and infection with *Sp*. P2431 on day 8 of life, pneumococcal shedding was monitored daily from day 1-4 p.i. (**Figure 1A**). Shedding values were significantly higher on day 2 p.i. in CSE-treated pups compared to PBS-treated pups (**Figure 2A**, *p*=0.030). Comparing the total number of shed *Sp*. from days 1-4 p.i. shows a significant increase in the number of *Sp*. shed in the mice treated with CSE over those which received PBS (**Figure 2B**, *p*=0.0041). Based on the previous report showing that over 300 CFU/20 taps of shedding were required for effective pneumococcal transmission (Zafar et al., 2016), we evaluated the number of events from days 1-4 p.i. that shedding were over 150 CFU/10 taps and the ratio of positive events was significantly higher in CSE-treated group by Fisher’s exact test (*p*=0.031). As previous reports have shown suggestions of a positive correlation between pneumococcal shedding and local neutrophil recruitment in the nasal cavity (Richard et al., 2014; Kono et al., 2016; Zafar et al., 2016), we performed flow cytometry with the nasal lavages obtained on day 2 p.i. from CSE or PBS pre-treated pups as this was the day when the bacterial shedding was significantly different between both groups (**Figure 2C**). There were significantly higher numbers of neutrophils in the nasal cavity of the CSE group on day 2 p.i. (*p*=0.0014). Together, these results suggest that passive smoking increases pneumococcal shedding early in infection by inducing increased inflammation in the URT mucosa and a higher density of colonizing bacteria.

### Cigarette Smoke Exposure Promotes Host-to-Host Pneumococcal Transmission

**Figure 3:**
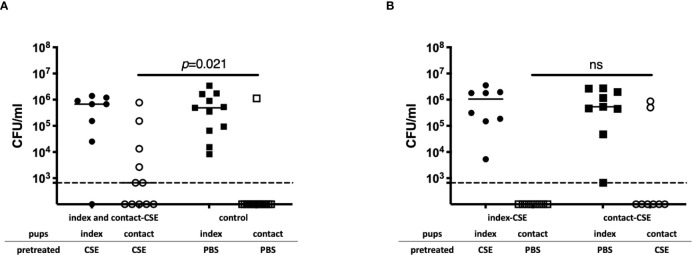
Pneumococcal transmission with or without CSE. **(A)** Pneumococcal colonization of index and contact mice in the index and contact-CSE group (index and contact received CSE) (*n* = 14) and the control group (index and contact received PBS) (*n* = 11). **(B)** Pneumococcal colonization of index and contact mice of index-CSE group (*n* = 8) and the contact-CSE group (*n* = 8) transmission studies. Each symbol represents the colonization density in nasal lavages for individual pups (CFU/ml), with median values indicated by a bar. CSE group (circles), PBS group (square); index mice (closed symbols), contact mice (open symbols). Dotted line indicates the limit of detection (666 CFU/ml). Mann-Whitney U test was used for the statistical analyses.

To evaluate the impact of passive smoking on pneumococcal transmission, we compared transmission of *Sp*. among index and contact-CSE group (both index and contact pups were CSE-treated) to transmission within PBS-treated litters (control group). The density of *Sp*. colonization among contact pups in the index and contact-CSE group was significantly higher than the control group (*p*=0.021; **Figure 3A**) and the transmission rate among the CSE-treated group (55%) was also significantly higher compared to the PBS-treated litters (7%) by Fisher’s exact test (*p*=0.021; **Table 1**). Next, to evaluate whether shedding or acquisition is the factor more influenced by cigarette smoke exposure, we assessed transmission within litters in which only the index pups received CSE (index-CSE group) compared to transmission in litters in which only the contact pups received CSE (contact-CSE group; **Figure 3B** and **Table 1**). There were no transmission events observed in the index-CSE litters, and in the contact-CSE groups the transmission rate was 25%; however, there were not any significant differences compared to the control group (in which both index and contact mice received PBS; **Table 1**). These results suggest that exposure of CSE in both the donor (index) and recipient (contact) pups increases rates of pneumococcal transmission.

**Table 1:** Summary of pneumococcal transmission experiments. Transmission rates of each group were compared to control group by Fisher’s exact test.

Group	Treatment of index	Treatment of contact	Number of contacts colonized/total	Transmission rate	Fisher’s exact test compared with control group
control group	PBS	PBS	1/14	7.1%	–
Index and contact- CSE group	CSE	CSE	6/11	54.5%	*p*=0.021
Index-CSE group	CSE	PBS	0/8	0%	not significant
Contact-CSE group	PBS	CSE	2/8	25%	not significant

### Cigarette Smoke Exposure Enhances Susceptibility to Pneumococcal Acquisition

**Figure 4:**
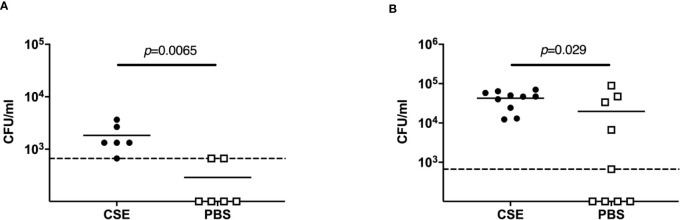
Acquisition model. Nasopharyngeal pneumococcal colonization assessed by nasal lavages 7 hours **(A)** and 24 hours **(B)** post-infection with ~1,000 CFU *Sp*. 6A. Median values are indicated by a bar and each symbol represents the CFU/ml of a single mouse. CSE group (black circle) (*n* = 6 in 7 hours p.i. and 10 in 24 hours 4 p.i.) and PBS group (open square) (*n* = 6 in 7 hours p.i. and 9 in 24 hours 4 p.i.). Dotted line indicates the limit of detection (666 CFU/ml). Mann-Whitney U test was used for the statistical analyses.

To investigate how passive smoking influences initial pneumococcal colonization, we examined upper respiratory tract colonization density at early points after intranasal infection. For this experiment, we infected pups with a lower dose of *Sp*. to more closely model natural acquisition during transmission experiments. We chose a bacterial dose of >1,000 CFU as the total *Sp*. shed from a single colonized mouse in the CSE-treated group over 4 days was estimated at ~1,000 CFU (**Figure 2B**). In pups given 1000 CFU intranasally after CSE treatment, there were significantly more bacteria at 7 hours (*p*=0.0065) and 24 hours (*p*=0.029) post-infection (**Figures 4A, B**) compared to pups that were pre-treated with PBS. On the other hand, there could not find statistical difference in the bacterial density in the nasal cavity at day 4 post-infection (data not shown). This suggests that passive smoking enhances susceptibility to an initial step of colonization by pneumococcus in a new host.

### Nasopharyngeal Inflammation by Cigarette Smoke Exposure Causes Disruption of the Mucosal Barrier and Increased Pneumococcal Attachment

**Figure 5:**
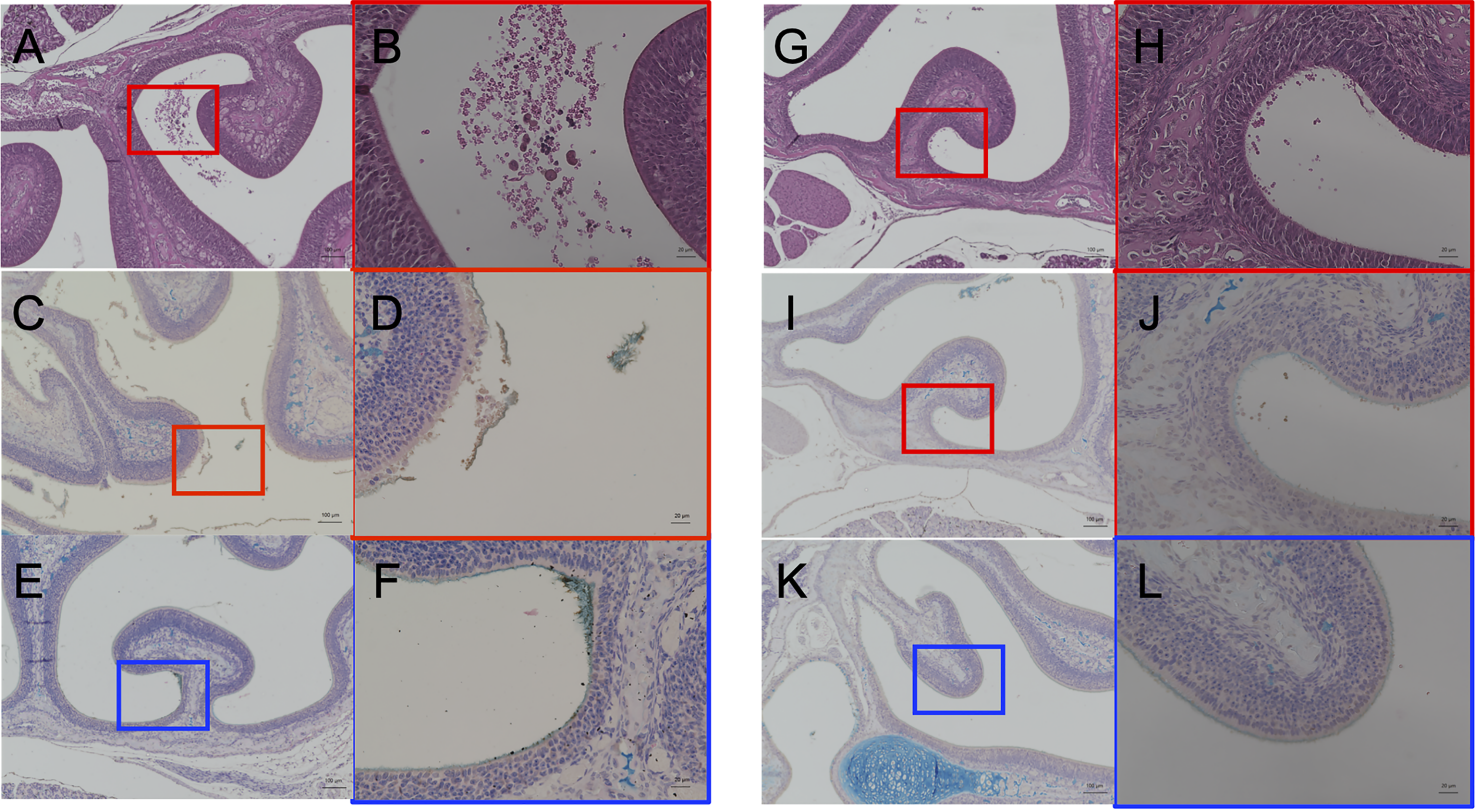
Histological analyses of the nasal cavity of pups by hematoxylin and eosin staining **(A, B, G, H)** and immunohistochemistry double staining of type 6A pneumococcal capsule polysaccharide and Alcian blue for mucus **(C–F, I–L)**. CSE pre-treated group; **(A–F)**, PBS pre-treated group; **(G–L)**. **(A, C, E, G, I, K)** show low-magnification field (100×magnification. Scale bar, 100 µm) and **(B, D, F, H, J, L)** are high-magnification field of boxed sections (400×magnification. Scale bar, 20µm). Three mice for each group were examined for the analyses and the representative images were displayed.

At day 2 p.i., when the shedding of the CSE-treated group was significantly higher than the PBS group (**Figure 2A**), we performed a histological examination of the nasal tissue from CSE and PBS pre-treated pups. Representative images are shown in **Figure 5**. Following staining of nasopharyngeal tissue sections with hematoxylin and eosin (HE), we observed more damaged tissue and vascular leakage (hemorrhage and inflammatory cells) in the nasal cavity of the mice pre-treated with CSE than mice treated with PBS (**Figures 5A, B** compared to **5G, H**). In sections which were stained for the pneumococcal capsule by anti-polysaccharide-specific sera and for mucus by Alcian blue, we identified clusters of *Sp*. surrounded by mucus (**Figures 5E, F**) and disruption of the mucosal membrane, as well as adhesion of the pneumococcus to exposed submucosa in the CSE-treated pups (**Figures 5C, D**). In contrast, the disruption of the mucosal membrane and luminal bacteria were fewer in the PBS group, although the bacteria were still observed along the undisrupted surfaces of the nasal cavity (**Figures 5I–L**). These findings suggest CSE causes tissue damage in upper respiratory tract which appears to facilitate attachment of the pneumococcus to the host mucosal surface.

### Cigarette Smoke Exposure Induces Mucus Secretion in the Nasopharynx

**Figure 6:**
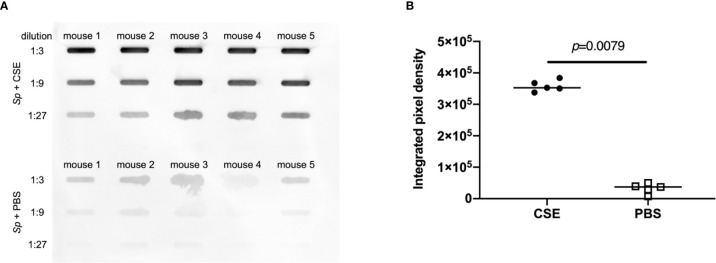
Comparison of sialic acid secretion. Nasal lavages were analyzed by slot-blot immunoblot for α-2,3-linked sialic acids by MAL-II binding. **(A)** Each band represents MAL-II binding to lavages from individual mice. **(B)** The relative intensities of the bands on the immunoblot were quantified by measuring the integrated pixel density (IPD). Bar represents the median and each symbol represents IPD from an individual band. CSE group (black circle) and PBS group (open square) (*n* = 5 in each group). Mann-Whitney U test was used for the statistical analyses.

To assess the effect of CSE on mucus production in the upper respiratory tract, we quantified sialic acid levels in the nasal lavages by immunoblotting, probing with Maackia Amurensis Lectin II (MAL II), which binds α-2,3-linked sialic acids. We wanted to assess the relationship between CSE treatment and mucus secretion under “acquisition model” conditions (infecting with a low-dose of *Sp*.). After pre-treatment with CSE or PBS (**Figure 1A**), pups were infected with ~1,000 CFU *Sp*.; nasal lavages were obtained 24 hours p.i. The levels of sialic acid were significantly increased in the URT lavages obtained from the CSE-treated group compared to the PBS-treated group (**Figures 6A, B**), suggesting that mucus secretion is elevated by passive smoking in the setting of pneumococcal acquisition in the early phase of infection.

## Discussion

In this report, we demonstrate that cigarette smoke exposure promotes host-to-host transmission of *S. pneumoniae* in a neonatal mouse model by enhancing pneumococcal shedding from the colonized host and by increasing susceptibility to infection in a new host. Previous studies revealed that an inflammatory environment in the upper respiratory tract, caused by co-infection with influenza A virus, was an important factor in promoting colonization and transmission of the pneumococcus (Short et al., 2012; Richard et al., 2014; Kono et al., 2016; Zafar et al., 2016). In particular, co-infection with influenza virus significantly increased bacterial load and neutrophil migration to the nasopharynx, which resulted in a significant increase in bacterial shedding compared to mice that were infected with *Sp*. alone. We have adapted this transmission model to evaluate the effect of cigarette smoke exposure on the donor and recipient by treating pups with CSE in lieu of co-infection with influenza virus.

In the transmission process, both shedding from a colonized host and acquisition in new host are critical factors. In the current study, CSE treatment affected both donor (index) and recipient (contact) pups: the effects of CSE treatment on the index pups include 1) the induction of inflammation in the nasal cavity, 2) increased pneumococcal burden, and 3) increased shedding. Differences were observed in colonization and bacterial shedding between CSE and PBS exposed group. Considering CSE exposure was required to both index and contact group to enhance transmission, this increase of shedding was an important factor for index side in this model. In the CSE-treated group, the increase in pneumococcal shedding positively correlated with the influx of neutrophils to the nasal cavity. Previous reports have shown that pneumococci aggregate with neutrophils that have migrated to the nasal cavity and are then secreted out the nose (Richard et al., 2014) which concurs with the results described in this study. Furthermore, examination of nasopharyngeal tissue sections revealed mucosal epithelial damage of the nasal cavity due to CSE treatment and *Sp.* infection, and, interestingly, we noted many pneumococci adhered to the shed mucosal epithelium. This finding may suggest that the shed mucosal epithelium plays the role of a vehicle that carries pneumococci out of the nose. The expression of mRNA of cytokines (IL-1α, IL-1β, IL-6 and TNF-α) in the nasal lavages were measured by qPCR to evaluate the level of inflammation. But the levels of mRNA expression were low in all pups suggesting limitations of the quantitative evaluation of cytokines in infant upper respiratory tract (data not shown). We showed a significant increase in sialic acid-containing mucus secretions in the nasal washes of the *Sp*.+CSE group compared to *Sp*.+PBS pups by slot blot assay. Together with the pathology findings, this suggests that increased nasal mucus is an important factor responsible for the increased shedding of pneumococci by CSE treatment. Cigarette smoke itself has been reported to enhance mucin (*Muc5AC* and *Muc5b*) expression in the respiratory tract and to promote mucus secretion (Shao et al., 2004; Ueha et al., 2017; Cao et al., 2018).

In previous reports, factors which affect the donor (index pups) and subsequently influence pneumococcal transmission have been well-examined, but the investigation on the recipient (contact pup) side of the process has been lacking. In one of the few studies which examined the role of acquisition, it was shown that when contact pups were immune to *Sp*., transmission was completely prevented, suggesting that immunity before exposure is critical for protection of a new host (Zangari et al., 2017). In this study we also examined the impact of CSE on recipient (contact) pups. CSE-treated pups showed increased *Sp*. colonization in the nasopharynx at early timepoints post-inoculation compared to PBS pups, suggesting that susceptibility to pathogens may be increased. As it is known to provide protection against microbial colonization, we examined the integrity of the mucosal barrier after CSE treatment in *Sp.*-infected mice. In these pups we found that the mucus and epithelial layers had sloughed off, and it appears this creates a favorable environment for the pneumococcus to attach on the mucosal surface and establish colonization. The reports that, in humans, cigarette smoking increases platelet-activating factor receptor (PAFR) expression and stimulates PAFR-dependent adhesion of the pneumococcus to airway epithelial cells would also be an explanation contributing to the increased susceptibility of these pups (Cundell et al., 1995; Grigg et al., 2012; Shen et al., 2016).

The increased pneumococcal shedding and increased susceptibility to pneumococcal colonization of contact pups due to CSE treatment were both found to be required factors for increased pneumococcal transmission, as no significant increase in transmission was observed when CSE was administered to only the index or contacts pups. Passive smoking is a major public health problem for children, and this study has demonstrated that cigarette smoke exposure promotes pneumococcal colonization and transmission. It is considered that the prevention of passive smoking is effective for prevention of pneumococcal transmission for both *Sp*. carriers and non-carriers, and has high social significance.

The primary limitation of this study is that pups received cigarette smoke by liquid extract (CSE) to avoid the influences of inhalation of cigarette smoke on the dam. CSE is a product in which a mainstream of smoke is dissolved in saline. Although the differences of direct inhalation of cigarette smoke and topical administration of CSE are difficult to evaluate, published reports on the use of CSE inoculation into the respiratory tract of animal models conclude that the results did not conflict when compared to results due to direct inhalation of cigarette smoke (Elliott et al., 2006; Mizutani et al., 2009; Wu et al., 2014; Ueha et al., 2016; Jia et al., 2019). It is difficult to quantify passive smoking and the total exposure may not correlate with the number of cigarettes. When interpreted the current model of mice into human the dose of CSE, while its relatively a lot amount, would be reliable and our model reflects a situation close to the passive smoking in the real life by considering sidestream smoke contains much more chemical component and is thought to be more harmful than mainstream smoke (World Health Organization, 2010).

This is the first study which reports that cigarette smoke exposure promotes pneumococcal host-to-host transmission in a neonatal animal model. Experience from the use of conjugate pneumococcal vaccines has demonstrated that young children are a main source of transmission in the community. Our data suggest the avoidance of passive smoke exposure among children may decrease overall pneumococcal transmission, the first step of invasive pneumococcal disease.

## Data Availability Statement

The original contributions presented in the study are included in the article/supplementary material. Further inquiries can be directed to the corresponding author.

## Ethics Statement

The animal study was reviewed and approved by the Institutional Animal Care and Use Committee at Wakayama Medical University.

## Author Contributions

MK, JNW and MH conceived and designed the experiments. MD, MK, DN and FK performed the experiments. MD, MK, TZ and YM analyzed the data. MD, MK and MH wrote the paper. All authors contributed to the article and approved the submitted version.

## Funding

This project was supported by grants from Smoking Research Foundation (No. N101) to MK and GlaxoSmithKline Japan Research Grant 2018 (No. C-34) to DM. The funder was not involved in the study design, collection, analysis, interpretation of data, the writing of this article or the decision to submit it for publication.

## Conflict of Interest

The authors declare that the research was conducted in the absence of any commercial or financial relationships that could be construed as a potential conflict of interest.
